# Exploring the Polyadenylated RNA Virome of Sweet Potato through High-Throughput Sequencing

**DOI:** 10.1371/journal.pone.0098884

**Published:** 2014-06-05

**Authors:** Ying-Hong Gu, Xiang Tao, Xian-Jun Lai, Hai-Yan Wang, Yi-Zheng Zhang

**Affiliations:** 1 College of Life Sciences, Sichuan University, Key Laboratory of Bio-resources and Eco-environment, Ministry of Education, Sichuan Key Laboratory of Molecular Biology and Biotechnology, Center for Functional Genomics and Bioinformatics, Chengdu, Sichuan, People’s Republic of China; 2 Chengdu Institute of Biology, Chinese Academy of Sciences, Chengdu, Sichuan, People’s Republic of China; Institute of Infectious Disease and Molecular Medicine, South Africa

## Abstract

**Background:**

Viral diseases are the second most significant biotic stress for sweet potato, with yield losses reaching 20% to 40%. Over 30 viruses have been reported to infect sweet potato around the world, and 11 of these have been detected in China. Most of these viruses were detected by traditional detection approaches that show disadvantages in detection throughput. Next-generation sequencing technology provides a novel, high sensitive method for virus detection and diagnosis.

**Methodology/Principal Findings:**

We report the polyadenylated RNA virome of three sweet potato cultivars using a high throughput RNA sequencing approach. Transcripts of 15 different viruses were detected, 11 of which were detected in cultivar Xushu18, whilst 11 and 4 viruses were detected in Guangshu 87 and Jingshu 6, respectively. Four were detected in sweet potato for the first time, and 4 were found for the first time in China. The most prevalent virus was SPFMV, which constituted 88% of the total viral sequence reads. Virus transcripts with extremely low expression levels were also detected, such as transcripts of SPLCV, CMV and CymMV. Digital gene expression (DGE) and reverse transcription polymerase chain reaction (RT-PCR) analyses showed that the highest viral transcript expression levels were found in fibrous and tuberous roots, which suggest that these tissues should be optimum samples for virus detection.

**Conclusions/Significance:**

A total of 15 viruses were presumed to present in three sweet potato cultivars growing in China. This is the first insight into the sweet potato polyadenylated RNA virome. These results can serve as a basis for further work to investigate whether some of the 'new' viruses infecting sweet potato are pathogenic.

## Introduction

The sweet potato [*Ipomoea batatas* L. (Lam.)] originated in South America and was transported across the pacific by Polynesians [1]. It has been cultivated by humans for up to 8,000 years, and today it is widely grown around the world due to its strong adaptability, easy management, rich nutrient content and multiple usages. Sweet potato is the fifth most important food crop in developing countries. About 130 million metric tons of tuberous roots are produced globally each year on about 9 million hectares of land [2], [3]. China is the biggest producer in the world, accounting for 80% of the global sweet potato production [4]. Compared to other staple food crops sweet potato needs fewer inputs, but produces more biomass [5]. A few researchers have shown interest in sweet potato mainly because of its complex hexaploid inheritance [6]. Recently, the growing awareness of health benefits attributed to sweet potato has stimulated renewed interest in this crop [3].

Viral diseases are the second most significant biotic stress for sweet potato after the sweet potato weevil [7]. Usually, sweet potato viruses will co-infect the plants and severely limit root production [8]. Yield losses caused by these viral diseases reach 20% to 40%, but this can reach near 100% in some African countries [9]–[12]. Over 30 viruses have been reported to infect sweet potato worldwide, but most of them are asymptomatic [3], [13]. Eleven of these viruses have been detected in China [14], including *Sweet potato C6 virus* (SPC6V) [15], *Sweet potato chlorotic fleck virus* (SPCFV) [15], *Sweet potato chlorotic stunt virus* (SPCSV) [15]–[17], *Sweet potato collusive virus* (SPCV, synonym Sweet potato caulimo-like virus) [10], *Sweet potato feathery mottle virus* (SPFMV) [15], [18]–[20], *Sweet potato leaf curl virus* (SPLCV) [21], *Sweet potato latent virus* (SwPLV) [19], [20], *Sweet potato mild mottle virus* (SPMMV) [15], *Sweet potato mild speckling virus* (SPMSV) [15], *Sweet potato virus G* (SPVG) [20], [22] and *Sweet potato virus 2* [SPV2, synonym Ipomoea vein mosaic virus (IVMV) or Sweet potato virus Y (SPVY)] [23], [24]. SPFMV, SwPLV and SPCFV were recognized to be the most commonly occurring and damaging viruses in China [4], [14]. Infection rates of these three viruses in major production regions of China range from 21% to 100% [15], [17], resulting in annual economic losses of about $639 million to the Chinese sweet potato industry [25].

Up until 1995, most of the work on sweet potato virus focused on SPFMV, but in the past 18 years, due to the advent of molecular biology, various comprehensive studies on virus composition and the effects of viral diseases were reported [3], [26]–[28]. The development of next-generation sequencing (NGS) technology provides a highly sensitive method for virus detection and diagnosis [29]–[31]. In this study, we analyzed the NGS data of eight sweet potato tissues and re-analyzed those of the other two published studies [32], [33] to identify RNA virus sequences.

## Materials and Methods

### Plant Material

Sweet potato plants (*I. batatas* cv. Xushu 18) were planted in an experimental field at Sichuan University under natural conditions. All of the following samples were collected from symptomless plants: Fibrous roots (FR) at one month after planting; young leaves (YL), mature leaves (ML), stems and initial tuberous roots (ITR) at 1.5 months; expanding tuberous roots at 3 months; harvested tuberous roots at 5 months; newly opened flowers were collected from symptomless drought-treated plants at 4 months.

### High-throughput RNA-sequencing

Total RNA was extracted using the TRIzol Reagent (Invitrogen), and genomic DNA removed with DNase I (Fermentas, Burlington, Ontario, Canada) according to the manufacturer’s instructions. Then the purity, concentration and RNA integrity number (RIN) of total RNA were measured with a SMA3000 and/or Agilent 2100 Bioanalyzer. The assessed total RNA was submitted to the Beijing Genomics Institute (BGI)-Shenzhen, Shenzhen, China (http://www.genomics.cn) for mRNA purification and RNA sequencing (RNA-Seq) with Illumina Hiseq 2000.

### Viral Sequence Mining and Expression Pattern Analyses

To investigate the polyadenylated RNA virome of sweet potato, viral sequences and expression patterns were mined from the vegetative transcriptome of Xushu 18 according to the annotation information and Digital Gene Expression (DGE) profiling results [34]. We also extracted total RNA from floral organs of sweet potato cultivar Xushu 18 and submitted it to Illumina HiSeq 2000 for RNA-Seq analysis [35]. By using Bowtie [36] under default parameters except seed length of 40 and mismatches of 3, the 90 bp paired-end (PE) reads of the floral organs were mapped to the vegetative transcriptome, that has been known to contain some viral sequences. Moreover, the 75 bp PE reads of Guangshu 87 [32] and Jingshu 6 [33] retrieved from the NCBI’s Sequence Read Archive database (http://www.ncbi.nlm.nih.gov/Traces/sra) (Table 1) were re-analyzed by using Bowtie [36] to align them to the vegetative transcriptome. The number of mapped read pairs or tags was counted according to the mapping results. RPKM (Reads Per Kilobase per Million mapped reads) [37] and TPM (Transcripts Per Million clean tags) [38] were calculated and used for quantifying each viral transcript in different sweet potato samples.

**Table 1:** Summary of RNA-Seq and DGE data used in this study.

Accession No.	Cultivar	Planting location	Tissue types	No. of tags or read pairs	References	Note
SRA043582	Xushu 18	Chengdu	Leaves, stems and roots	48,716,884	[34]	Sequencing directly
SRA043584	Xushu 18	Chengdu	Flowers	41,533,336	[35]	Sequencing directly
SRA022988	Guangshu 87	Guangzhou	Roots	59,233,468	[32]	Re-analyze
SRA044884	Jingshu 6	Beijing	Roots	25,888,888	[33]	Re-analyze
GSE35929	Xushu 18	Chengdu	Young leaves	3,352,753	[34]	Sequencing directly
GSE35929	Xushu 18	Chengdu	Mature leaves	3,429,018	[34]	Sequencing directly
GSE35929	Xushu 18	Chengdu	Stems	3,453,654	[34]	Sequencing directly
GSE35929	Xushu 18	Chengdu	Fibrous roots	3,583,907	[34]	Sequencing directly
GSE35929	Xushu 18	Chengdu	Initial tuberous roots	3,630,619	[34]	Sequencing directly
GSE35929	Xushu 18	Chengdu	Expanding tuberous roots	3,566,630	[34]	Sequencing directly
GSE35929	Xushu 18	Chengdu	Harvested tuberous roots	3,514,272	[34]	Sequencing directly

### Reverse Transcription Polymerase Chain Reaction (RT-PCR) Verification

Equal RNA extracted from FR, YL, ML, ITR and stems were reversely transcribed with Moloney murine leukemia virus (MMLV) reverse transcriptase (Invitrogen, Carlsbad, California, CA) using Oligo(dT) as primer. The resulting cDNA was subjected to viral sequence amplification and viral gene expression level analysis.

Fourteen pairs of primers were designed according to the assembled viral transcripts (Table 2) using Primer Premier 5.0 (PREMIER Biosoft. International, CA, USA) (Table 3), and sequence amplification was implemented using KOD-FX (TOYOBO, Osaka, Japan). The purified PCR products were sequenced with an ABI 3730 instrument to confirm the amplified sequences.

**Table 2:** Statistics of viruses found in sweet potato transcriptome annotations, estimated gene expression abundance.

Virus name	Abbreviation	Genus	No. of Sequences	Total length (bp)	Identity (%)	Average expression levels (RPKM)	No. of read pairs
*Sweet potato feathery mottle virus*	SPFMV	*Potyvirus*	47	22,302	100	50.99	46,434
*Sweet potato virus G*	SPVG	*Potyvirus*	5	2,781	100	14.79	1,680
*Northern cereal mosaic virus*	NCMV-like	*Cytorhabdovirus*	1	1,134	46	11.58	536
Sweet potato virus B2	SPVB2	*Potyvirus*	6	987	97	3.85	155
*Sweet potato latent virus*	SwPLV	*Potyvirus*	3	806	100	1.73	57
Sweetpotato badnavirus B	SPBV-B	*Badnavirus*	4	575	94	1.11	26
*Sweet potato virus 2*	SPV2	*Potyvirus*	3	419	100	1.40	24
Sweetpotato badnavirus A	SPBV-A	*Badnavirus*	2	250	97	1.57	16
Mikania micrantha mosaic virus	MMMV	*Fabavirus*	5	736	100	1.25	42
*Yam mosaic virus*	YMV	*Potyvirus*	1	230	94	5.11	48
*Turnip mosaic virus*	TuMV-like	*Potyvirus*	1	217	69	0.68	6
*Sunflower mosaic virus*	SuMV	*Potyvirus*	1	198	96	1.36	11

No. of Sequences means the viral transcripts detected in the vegetative transcriptome [34]. Total length means the sum of detected viral transcripts of each virus. Identity refers to sequence identities when similarity BLASTX search was conducted between assembled viral transcripts and NR database. YMV was further confirmed to be SPVG, and TuMV-like and SuMV were further confirmed to be SwPLV.

**Table 3:** Primers used for virus fragment amplification.

No.	Virus name	Primer name	Primer sequence (5′–3′)	Product length (bp)
1	SPVC	SPVC-CP1F	TCGGTGTATCATCAATCTGGC	560
		SPVC-CP1R	CCATCCATCATCGTCCAAAC	
2	SPFMV	SPFMV-SP1F	CTCCACCACCCACAATAACTG	402
		SPFMV-SP1R	TCCCCATTCCTGTATCGTCA	
3	SPVB2	SPVB2-P3F	GAGACAGCAGAAACAGCAGTGATA	471
		SPVB2-P3R	GGCATCACAATAAACCCATCCT	
4	SPVG	SPVG-1F	ACAACGTGCATCATCAGTCT	459
		SPVG-1R	CATTTGCCATTGGTGCTCTT	
5	SPVB2	SPVB2-P1F	AAGCATGTGGTGAAAGGAAAGTG	361
		SPVB2-P1R	TTGCTTGTTCATCCATTCCCTC	
6	SPVG	SPVG-2F	CGCCAACTAATAGCGAACTCT	549
		SPVG-2R	ACTATACGTCCATTCGCCATC	
7	SPBV-B	SPBV-C2PF	CAGGATTCACTCAGCAGACG	352
		SPBV-C2PR	ATGTCATGAAGGCACCTTCC	
8	SPBV-A	SPBV-C1PF	CAGCTTTGGTTGCTCTGCTATTT	477
		SPBV-C1PR	AAGACGGTTGGCCCATTGATAT	
9	SPV2	IVMV-P1F	TGCTGAAATGGGCATACTCC	489
		IVMV-P1R	TGCACACCTCTCATTCCTAACA	
10	SwPLV	SwPLV-P1F	CGAAGTGGATGACCAGCAGAT	500
		SwPLV-P1R	GGATTCCACGCATTCCAAGTAG	
11	NCMV-like	IbRNLS-PF	TCACCACAGAGGTACAAAGGAAA	1317
		IbRNLS-PR	ACCATGATTTACATCTCTGTCGG	
12	MMMV	MMWV2-P1F	ATGGTTGAATGCTCCCAAGACA	499
		MMWV2-P1R	CTCTCCATCCAATTCCCACCTAT	
13	SPLCV	SPLCV-P1F	GAAGCTATGTCCCGGTTTCAAGAG	300
		SPLCV-P1R	GCCTTCTGTCACGAATCAACCA	
14	CymMV	CymMV-P1F	CCTGAGCCCTTCTGTACCATA	775
		CymMV-P1R	GTGTTGGTGGAGCCAAGATG	

## Results

### Virus Identification *via* Next-generation Sequencing

Seven vegetative tissues were collected from sweet potato cv. Xushu 18 and equal RNA of each tissue sample was pooled together for RNA-Sequencing. A total of 48,716,884 PE reads were generated by Illumina/Solexa Genome Analyzer II. The *de* *novo* assembly and sequence annotation information were deposited at the Center for Functional Genomics and Bioinformatics of Sichuan University (http://cfgbi.scu.edu.cn/index.html). All of the results described above have been published in 2012 [34]. Sequences of nine viruses were detected in the vegetative organs of this cultivar (Table 2, Table S1). Among these viruses, two belonged to the *Badnavirus* genus: SPBV-A (sweet potato badnavirus A) and SPBV-B (sweet potato badnavirus B) which were suggested to be Sweet potato pakakuy virus (SPPV) (International Committee on Taxonomy of Viruses, ICTV, http://www.ictvonline.org). The others were all RNA viruses, in which SPFMV, SPVG, SwPLV, SPV2 and SPVB2 (sweet potato virus B2), YMV (*Yam mosaic virus*), TuMV-like (*Turnip mosaic virus*) and SuMV (*Sunflower mosaic virus*) are from the *Potyvirus* genus; and NCMV-like (*Northern cereal mosaic virus*) and MMMV (Mikania micrantha mosaic virus) are from *Cytorhabdovirus* and *Fabavirus*, respectively. MMMV is also known as Mikania micrantha wilt virus (MMWV) as it was first discovered in *Mikania micrantha* [39]. Furthermore, the results demonstrated that SPFMV and SPVG had the longest total sequence length, the highest mapped reads number and the highest average expression levels (Table 2). Except for SPFMV, SPVG, SwPLV and SPV2, the others were reported in sweet potato in China for the first time.

Sequence alignment analyses demonstrated that the SPFMV transcripts belonged to at least three SPFMV strains in this sweet potato cultivar, including the severe, common and ordinary strains [40]. The common strain had been renamed as *Sweet potato virus C* (SPVC) (ICTV, http://www.ictvonline.org). Furthermore, at least two distinct transcripts related to SPVG strains were identified (Table 4). According to the NGS annotation information, there were 3 short sequences s (230 bp, 217 bp and 198 bp, respectively) been annotated as YMV, TuMV-like and SuMV (Table 2). Further studies of the recent released genome sequences of SPVG and SwPLV have confirmed that the 3 short sequences were indeed from SPVG and SwPLV.

**Table 4:** Sequencing results of amplified viral fragments.

#	Length	Identities (%)^a^		Coverage^b^	E-value^c^	Viral names	GenBank Accession
	(bp)	A	B	(%)			Numbers
1	497	97.99	99	99	0	SPVC	JQ902097
2	358	98.88	99	100	3.00E-175	SPFMV	JQ902098
3	436	99.54	83	83	5.00E-97	SPVB2	JQ902099
4	394	98.48	99	100	0	SPVG-1	JQ902100
5	315	99.68	77	98	3.00E-60	SPVB2	JQ902101
6	510	98.24	78	99	4.00E-112	SPVG-2	JQ902102
7	308	99.25	77	99	7.00E-48	SPBV-B	JQ902103
8	438	99.58	94	23	0.004	SPBV-A	JQ902104
9	428	99.77	99	100	0	SPV2	JQ902105
10	462	95.64	96	98	0	SwPLV	JQ902106
11	981	99.80	39	88	4.00E-76	* IbRNLS	JQ902107

Sequence numbers are corresponded to Table 3;

a Sequence identities (A) were calculated between assembled and Sanger sequenced fragments; Sequence identities (B) were calculated between Sanger sequenced fragments and reference sequences from NCBI;

b, c Indicate the query coverage and E-value of Sanger sequenced segments BLAST against the reference sequences from NCBI;

*This is a Cytorhabdovirus-like sequence; the corresponding sequence identity (B), query coverage and E-value were obtained by TBLASTN searches.

Flowers of this cultivar were also collected and submitted to the NGS platform for RNA-Seq study. A total of 41,533,336 PE reads were generated [35]. By mapping these 90 bp PE reads to viral sequences retrieved from NCBI, and assembled viral sequences described above using Bowtie [36], we found four different virus-related transcripts belonging to SPFMV, SPVG, SPLCV and Cymbidium mosaic virus (CymMV). However, the mapped read number for them was only 6, 22, 3 and 20, respectively. These results indicated that fewer viral sequences presented in flowers than the vegetative organs. Of these, CymMV was found for the first time in sweet potato.

### Sequence Amplification by RT-PCR

**Figure 1:**
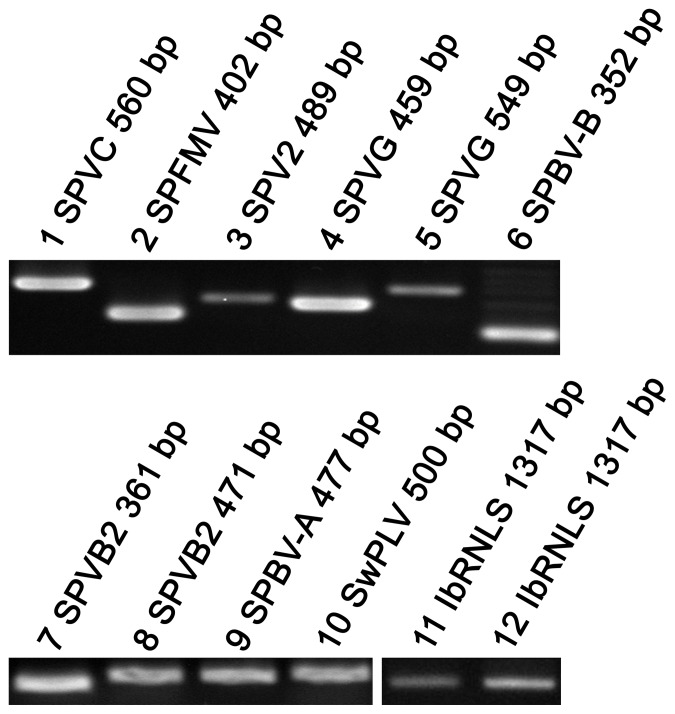
Electrophoresis of PCR products of amplified viral sequences. 1∼10 correspond to sequence numbers in Table 3 of 1 (560 bp), 2 (402 bp), 9 (489 bp), 4 (459 bp), 6 (549 bp), 7 (352 bp), 5 (361 bp), 3 (471 bp), 8 (477 bp) and 10 (500 bp), respectively. 11 and 12 correspond to sequence numbers in Table 3 of 11 (1317 bp) amplified from cDNA and genomic DNA, respectively.

RT-PCR analysis was conducted to verify whether all of these viral sequences existed in sweet potato cultivar Xushu 18. Fourteen pairs of primers were designed according to the assembled sequences (Table 3). Except MMMV, SPLCV and CymMV, 11 virus fragments of the expected sizes were successfully amplified from Xushu 18 (Figure 1). All amplified fragments were re-sequenced by the Sanger method and then were submitted for a sequence similarity search by BLASTN or TBLASTN. The results showed that all fragments had a high identity of ≥95% with the assembled sequences (Table 4). These indicated that deep sequencing technology could provide a reliable method to identify viral sequences. Two of the three SPFMV sequences identified by NGS showed 99% sequence identity with SPVC and the severe strain of SPFMV. Two SPVG sequences showed 99% and 78% identity with two different SPVG strains.

Comparing the re-sequenced fragments with the reference sequences retrieved from NCBI, the identities decreased for most of these amplified fragments, especially for the NCMV–like fragment (Table 4). The SPBV-B fragment shared 77% sequence identity with the reference sequence, and SPBV-A shared 94% identity with the reference in a short segment. These results indicated that SPBV-A and SPBV-B could perhaps be new sweet potato viruses. In this study we tentatively named them as Sweet potato badnavirus C (SPBV-C). Interestingly, the NCMV-like fragment failed to find a homologous nucleotide sequence from NCBI by BLASTN, so TBLASTN was employed to blast the deduced protein sequences to the translated nucleotide database. The results showed that it was homologous with Rhabdovirus N-like sequences (RNLSs) [41] and should be a new sweet potato virus. So we tentatively named it as *Ipomoea batatas* Rhabdovirus N-like sequences, IbRNLS.

### Confirmation of Four New Sweet Potato Viruses

**Figure 2:**
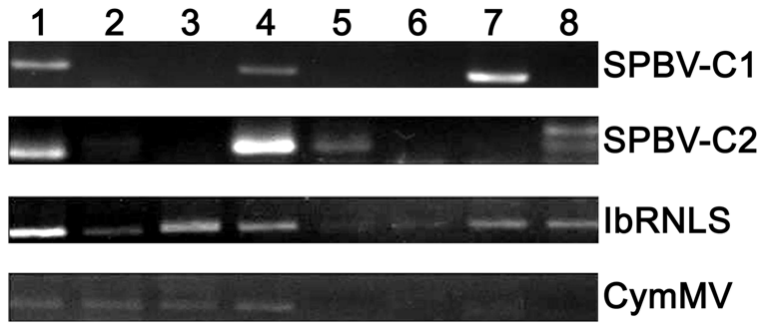
Amplification of four novel viruses from different sweet potato cultivars. 1∼8 correspond to sample numbers in Table 5.

To identify whether these four new virus-related sequences are present in other sweet potato cultivars in China, we collected eight different sweet potato tuberous root samples from different regions in Sichuan Province, China (Table 5). All of these eight sweet potato cultivars were cultivated by farmers under natural conditions. Total RNA was extracted for RT-PCR analysis. The results demonstrated that the SPBV-C1 viral sequence was amplified from three cultivars, SPBV-C2 was amplified from four cultivars, the NCMV-like presented itself in all eight tuberous root samples, and CymMV presented itself in four samples (Figure 2). These results confirmed that four new virus-related sequences are present in most of the sweet potato cultivars in this region.

**Table 5:** Sweet potato cultivars collected from different regions.

Sample No.	Cultivar	Source
1	Chuanshu 34	Sichuan academy of agricultural sciences, Chengdu City
2	Liyuan 1	Dayi County, Chengdu City
3	Nanshu 007	Dayi County, Chengdu City
4	Nanshu 88	Dayi County, Chengdu City
5	Unknown	Tianquan County, Ya’an City
6	Xushu 18	Weiyuan County, Neijiang City
7	Yusu 303	Dayi County, Chengdu City
8	Xushu 18	Zizhong County, Neijiang City

### Expression Patterns among Different Tissues

**Figure 3:**
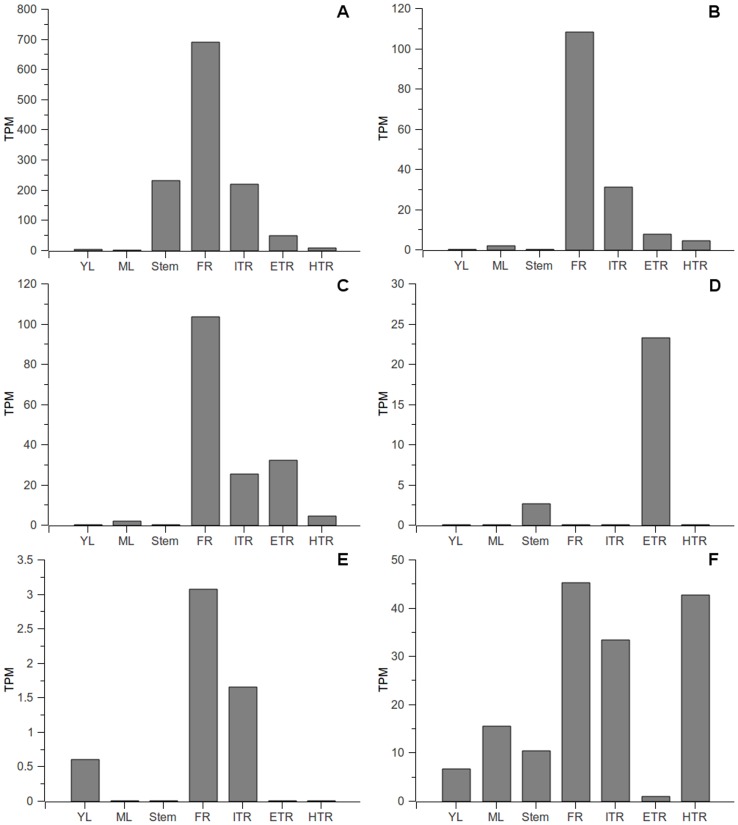
Digital gene expression analyses of different viral transcripts. TPM: Transcripts Per Million clean tags (Morrissy AS, 2009); YL, ML, Stem, FR, ITR, ETR and HTR indicate young leaves, mature leaves, stems, fibrous roots, initial tuberous roots, expanding tuberous roots and harvest tuberous roots, respectively; A∼F: indicate SPVC (Transcript_859), SPFMV (Transcript_11), SPVG (Transcript_5373), SPV2 (Transcript_81617), SwPLV (Transcript_95916) and NCMV-like (Transcript_5902), respectively.

DGE provides a new expression analysis method showing major advances in robustness, resolution and inter-lab portability over microarray and quantitative RT-PCR [42]. For this technology, 21 bp tags were sequenced for each mRNA; the tag number of each transcript gave a digital signal to characterize the expression patterns. To study the gene expression patterns of each virus transcript, all DGE tags from the seven vegetative tissues [34] (Table 1) were used for expression profiling. It was found that there were 16 transcripts containing a *Nla*III recognition site (CATG), which is the motif of DGE tags. These transcripts belonged to SPVC (5 transcripts), SPFMV (5 transcripts), SPVG (3 transcripts), SPV2 (2 transcripts), SwPLV (1 transcript) and NCMV-like (1 transcript). DGE quantification results showed that different viral transcripts had different expression levels (Figure 3). Transcripts of SPVC, SPFMV and SPVG had very high expression levels, which were about 100 times higher than that of the SwPLV transcript. These are consistent with the findings described above and illustrate that SPVC, SPFMV and SPVG may be the most prevalent viruses in China. Furthermore, it was found that all these viral transcripts were unevenly distributed in different tissues (Figure 3). Five of these six viruses possessed the highest expression level in fibrous roots, while the remaining one, in expanding tuberous roots. Initial tuberous roots also had a comparably high expression level for most of them, but young leaves, mature leaves and stem had lower expression levels. For example, SPFMV (Transcript_11) had an expression level of 107.97 TPM in fibrous roots and 31.12 TPM in initial tuberous roots, but only 1.75 TPM in mature leaves and no expression in young leaves. The highest expression levels of SPVC (Transcript_859) was also observed in fibrous roots (688.35 TPM), followed by stem (230.48 TPM) and initial tuberous roots (219.25 TPM).

**Figure 4:**
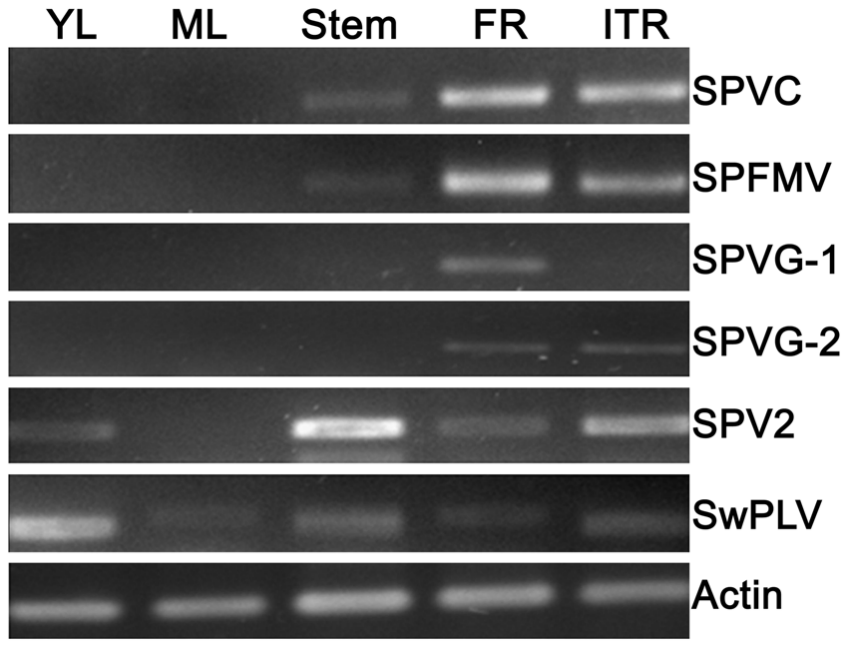
Virus gene expression levels among different tissues. YL, ML, Stem, FR, ITR, ETR and HTR indicate young leaves, mature leaves, stems, fibrous roots, initial tuberous roots, expanding tuberous roots and harvest tuberous roots, respectively.

By using sweet potato beta-actin as an internal control, expression levels of SPVC, SPFMV, SPVG, SPV2 and SwPLV transcripts were analyzed by semi-quantitative RT-PCR (Figure 4). For SPVC, fibrous and initial tuberous roots had almost equal expression levels, while young and mature leaves had the lowest levels. For SPFMV, highest expression levels were detected in fibrous roots, followed by initial tuberous roots, and no expression was observed in leaves. Similar expression patterns were also found for SPVG. But SPV2 and SwPLV had different expression patterns. The highest expression level was observed in stems for SPV2 and young leaves for SwPLV. However, for these two viruses, fibrous or initial tuberous roots also had relatively high expression abundance. There are some slightly differences of the relative expression levels among different tissues quantified by DGE and RT-PCR. But the reason for this discrepancy of the results between these two methods is unknown.

### Virus Identification in other Sweet Potato Cultivars

To investigate the polyadenylated RNA virome of the Guangshu 87 and Jingshu 6 cultivars, all the PE reads of their transcriptomes [32], [33] were aligned with viral sequences retrieved from NCBI, and the assembled sweet potato transcriptome. Results demonstrated that sequences of 11 virus species were found in cultivar Guangshu 87, including SPFMV, SPVG, SwPLV, SPLCV, SPCFV, SPVB1, SPVB2, SPVB3, SPBV-A, SPBV-B and CymMV. Sequences of 4 viruses were found in Jingshu 6, including SPFMV, SPVG, SPCFV and Cucumber mosaic virus (CMV). SPFMV and SPVG had the highest expression levels amongst all the viruses in these two cultivars (Table 6). Combining together all viruses identified from the three cultivars, we detected a total of 15 viruses, most of which were reported for the first time in China. Among these viruses, SPVC, SPFMV and SPVG had the highest expression levels in all three cultivars.

**Table 6:** Viral species detected by deep sequencing of other 3 sweet potato variety.

Virus name	No. of read pairs		
	Xushu 18	Guangshu 87	Jingshu 6
SPFMV	6	66,464	127
SPVG	22	9,332	511
SwPLV	0	126	0
SPLCV	3	12	0
SPCFV	0	1,055	17
SPVB1	0	12	0
SPVB2	0	36	0
SPVB3	0	5	0
SPBV-A	0	2	0
SPBV-B	0	22	0
CymMV	20	1	0
CMV	0	0	3

## Discussion

Sweet potato virus is usually detected using indicator plants such as *Ipomoea setosa*, *Ipomoea nil* and *Chenopodium quinoa* [10], [43], and electron microscopic observation [44], while molecular diagnosis is conducted using enzyme-linked immunosorbent assay (ELISA) [43], [45] or RT-PCR [18], [26], [46]–[48]. During the last decades, over 30 sweet potato viruses were detected in the world [3], [13], and 11 of these have been reported in China [14]. SPFMV, SPLV and SPCFV are considered as the major viruses in China [4], [14]. The advent of high-throughput sequencing technology offers a new and powerful approach for characterization of viruses. This methodology shows major advances in robustness, resolution and inter-lab portability [42]. It not only identifies known viruses, but also can identify low-titer and novel viral species without any prior knowledge [31].

In recent years, there were several groups investigating viral infection agents using high-throughput sequencing technology. For example, Kreuze *et* *al* successfully detected novel viruses from infected sweet potato and constructed complete viral genomic sequences by *de novo* assembling of 21 and 22 bp NGS reads [31], and Coetzee *et* *al* characterized the virome of a diseased South African vineyard [30]. In this study, transcripts of 11 virus species were identified in cultivar Xushu 18 through NGS data mining. For the vegetative transcriptome, 88% of the mapped viral PE reads were aligned to SPFMV or SPVC, and 87% of the rest were aligned to SPVG, these illustrated that SPFMV, SPVC and SPVG may be the most prevalent viruses in this cultivar. However, transcripts of only four virus species were detected in the flowers of Xushu 18, all of which had very low expression levels, which may indicate that viruses primarily accumulate in vegetative organs than in floral ones. Totally, transcripts of 15 viruses were identified from three sweet potato cultivars growing in China, four of which are novel sweet potato viruses, and several of which are reported in China for the first time.

Of the reported sweet potato viruses, most are associated with symptomless infections in sweet potato and in some cases even in the indicator plant. Some are synergized by SPCSV, the mediator of severe virus diseases in sweet potato, while others apparently are not [3]. Otherwise, sweet potato cultivars differ greatly in their reaction to the viruses, with some being symptomlessly infected, while others apparently immune [49]. The most common virus infecting sweet potato worldwide, SPFMV, can be symptomless, at least in some varieties [45], [50]. Previous research showed that nearly 70% of the symptomless plants were SPFMV-infected in a virus survey in Kenya [51]. In this study, although transcripts of 15 viruses were identified, no SPCSV related fragment was found. This may be the reason why so many virus fragments were detected from Xushu 18 but no symptom could be observed. Our results also indicated that most of the symptomless field-grown sweet potatoes were infected by several viruses. Usually, leaves are collected for virus detection in sweet potato [48], [52]. However, based on the DGE (Figure 3) and RT-PCR (Figure 4) analyses in this study, we found that expression levels of most virus transcripts were unevenly distributed in different tissues. Most virus transcripts possess extremely low expression levels in young and mature leaves, but higher expression levels in fibrous roots and initial tuberous roots. This indicated that using leaves as a test sample may give false negative results, while fibrous root should be the optimal choice for virus detection in this crop.

Sweet potato is vegetatively propagated from tuberous roots or vines, and farmers usually take vines for propagation from the farm year after year. If the sweet potato is infected with viruses, they will be transmitted to the next generation and accumulate in this crop, resulting in significantly decreased yields. The virus expression analyses results described in this study indicated that high expression levels of most viruses in fibrous and initial tuberous roots may be the main reason for the germplasm decline and production decrease. For sweet potato, adventitious roots develop at the nodes of a vine cutting, and then some of these roots change their growth pattern and develop into tuberous roots [53]. Depending on the number of fibrous roots that will be induced to form tuberous roots, sweet potato plants will yield either a high root production or a low number of tuberous roots [53]. High virus expression levels in fibrous roots will adversely affect the development of the root system and then result in tuberous root initiation failure. A well-developed root system is a prerequisite for healthy plant growth [14], [54]–[56] and is recognized as a key factor of high tuberous root yield [57]. The development failure of tuberous roots caused by virus infection will significantly decrease the total bio-mass production. Previous studies demonstrated that tuberous roots of virus-infected sweet potato form later and expand slower than virus-free ones [14], [54]. Compared with that of healthy plants, virus-infected plants have a significantly higher respiration rate and lower photosynthetic rate [57], [58], and are more easily infected by the fungal pathogens *Monilochaetes infuscans* and *Ceratocystis fimbriata*, and the nematode *Pratylenchus coffeae*. All these physiological characteristics will inevitably result in final yield loss.

## Supporting Information

Table S1 Sequences annotation of candidate viral sequences identified from the sweet potato transcriptome.
(XLSX)
